# Correlation between CT Abdominal Anthropometric Measurements and Liver Density in Individuals with Non-Alcoholic Fatty Liver Disease

**DOI:** 10.3390/medicina59030500

**Published:** 2023-03-03

**Authors:** Dragoș Constantin Cucoranu, Marian Pop, Raluca Niculescu, Vlad Vunvulea, Irina-Bianca Kosovski, Radu-Ovidiu Togănel, Eliza Russu, Adrian Vasile Mureșan, Răzvan-Andrei Licu, Anca Bacârea

**Affiliations:** 1 Department of Radiology, Mures County Emergency Hospital, 540136 Targu Mures, Romania; 2 ME1 Department, George Emil Palade University of Medicine, Pharmacy, Science and Technology of Targu Mures, 540142 Targu Mures, Romania; 3 Emergency Institute for Cardiovascular Disease and Transplant of Targu Mures, 540136 Targu Mures, Romania; 4 Pathology Department, Mures Clinical County Hospital, 540011 Targu Mures, Romania; 5 Pathophysiology Department, George Emil Palade University of Medicine, Pharmacy, Science and Technology of Targu Mures, 540142 Targu Mures, Romania; 6 Department of Anatomy, George Emil Palade University of Medicine, Pharmacy, Science and Technology of Targu Mures, 540139 Targu Mures, Romania; 7 Doctoral School of Medicine and Pharmacy, George Emil Palade University of Medicine, Pharmacy, Science and Technology of Targu Mures, 38 Gheorghe Marinescu Street, 540139 Targu Mures, Romania; 8 Clinic of Vascular Surgery, Mures County Emergency Hospital, 540136 Targu Mures, Romania; 9 Department of Vascular Surgery, George Emil Palade University of Medicine, Pharmacy, Science and Technology of Targu Mures, 540139 Targu Mures, Romania

**Keywords:** hepatic steatosis, anthropometric measurements, computed tomography, non-alcoholic fatty liver disease

## Abstract

*Background*: With a growing frequency, nonalcoholic fatty liver disease (NAFLD) is the most prevalent chronic liver disease worldwide. NAFLD has a strong correlation with other metabolic disorders, such as obesity, particularly abdominal obesity, even though the underlying causes or risk factors are not entirely understood. This study aims to investigate correlations between abdominal anthropometric measurements and the presence and intensity of liver steatosis as assessed by unenhanced computed tomography (CT). *Methods*: One hundred and nineteen patients (male/female, 66/53; mean age 54.54 +/− 12.90 years) underwent abdominal non–contrast-enhanced CT. CT images were examined to determine the attenuation of liver parenchyma, subcutaneous fat depth, and waist circumference (WC). *Results*: Among all patients, WC (r = −0.78, *p* < 0.0001), infraumbilical subcutaneous fat thicknesses (r = −0.51, *p* < 0.0001), right paraumbilical subcutaneous fat thicknesses (r = −0.62, *p* < 0.0001), and left paraumbilical subcutaneous fat thicknesses (r = −0.53, *p* < 0.0001) had a high inverse correlation with the liver attenuation values. The presence of T2D (OR: 2.40, *p* = 0.04), WC (OR: 11.45, *p* < 0.001), right paraumbilical (OR: 10.09, *p* < 0.001), left paraumbilical (OR: 2.81, *p* = 0.01), and infraumbilical (OR: 3.06, *p* = 0.007) were strongly independent predictors of NAFLD risk. Moreover, regarding the laboratory parameters, only the higher value of GGT (OR: 2.84, *p* = 0.009) is a predictor of NAFLD risk. *Conclusions*: Our data show that higher baseline values of all abdominal anthropometric measurements are correlated with liver attenuation and act as predictors of NAFLD risk.

## 1. Introduction

The accumulation of fat, mostly triglycerides, in hepatocytes without evidence of excessive alcohol intake or other conditions is known as non-alcoholic fatty liver disease (NAFLD), the most common chronic liver disease, with a globally estimated prevalence ranging between 13% and 32% across different areas and ethnic groups [1,2,3]. With decreasing hepatitis incidence and growing obesity, NAFLD will become the predominant cause of end-stage liver disease during the next few decades [4,5]. As a result, there is a growing interest in researching NAFLD.

The severity of NAFLD varies, ranging from simple steatosis to steatohepatitis, with the possibility of developing into fibrosis and cirrhosis [6,7,8]. NAFLD is recognized as a hepatic manifestation of the metabolic syndrome and is intimately linked to insulin resistance, dyslipidemia, visceral obesity, and type 2 diabetes [4]. A sedentary lifestyle and a high-calorie diet are two risk factors that are directly linked to an unhealthy lifestyle. The main objective of reducing weight has been recognized as the key method for managing NAFLD through adequate lifestyle adjustments [9]. The pathophysiology of NAFLD begins with insulin resistance, which leads to excessive fat storage in the liver. The liver is prone to a variety of insults, including adipokine dysregulation, lipid peroxidation, inflammatory reactions, hepatic fibrosis, oxidative stress, and apoptosis [10]. Patients with metabolic syndrome, and particularly diabetes, have a high prevalence of NAFLD [11,12]. It is estimated that 82% of people with NASH and 51% of those with NAFLD, respectively, are obese worldwide [2]. NAFLD has also been linked to hypothyroidism, colonic adenomas, polycystic ovarian syndrome, and neoplasms [11]. Early identification of NAFLD is crucial to prevent the condition from progressing to more severe stages since individuals with NAFLD have higher risks of cardiovascular and liver-related mortality [4,13].

Imaging or histological evidence of hepatic steatosis, as well as the lack of secondary causes, are required for the diagnosis of NAFLD. The gold standard for diagnosing NAFLD is a liver biopsy, although doing so would be expensive, unnecessary, and impractical for most patients [14]. CT (computed tomography) enables the quantitative measurement of hepatic steatosis by evaluating the liver attenuation value, which is represented by Hounsfield units (HU). Due to the fact that fat has a significantly lower attenuation value than soft tissue, the attenuation value of hepatic parenchyma decreases as hepatic steatosis develops and progresses [15].

Although the underlying causes or risk factors for NAFLD are not fully understood, there is a significant association with other metabolic disorders, including obesity, particularly abdominal obesity. The waist circumference (WC) is an anthropometric measurement of obesity that is associated with abdominal adiposity and is thought to be a significant predictor of diabetes and NAFLD development [1,16,17,18]. In order to ease the early identification of individuals most likely to develop NAFLD, numerous studies have focused on developing convenient and easy alternatives for epidemiological investigations and thorough public health surveillance [19,20]. To determine the risk of NAFLD in this circumstance, a variety of simple anthropometric markers, biochemical markers, and combinations of indicators have been proposed. Among them, the most reliable indicators for predicting NAFLD are obesity and lipid-related indicators, which are often utilized in epidemiological research [21].

This study’s hypothesis is that abdominal subcutaneous fat depth and WC may be significantly correlated with NAFLD. The objective of this study is to determine the correlations between abdominal anthropometric measurements and the presence and severity of liver steatosis as determined by non-contrast-enhanced CT.

## 2. Materials and Methods

### 2.1. Study Cohort

Between January 2020 and February 2021, a total of 119 adult patients at our institution who received abdomen non-contrast-enhanced CT were enrolled in the study. Exclusion criteria included persons less than 18 years of age, any known pre-existing liver disease (save for steatosis), missing laboratory values, positive serology for hepatitis B or C viruses, hemochromatosis, documented alcoholism, and drug-induced liver injury. There was no room for subpar images. Demographic characteristics (age and gender) as well as laboratory measurements were extracted from the data (total cholesterol, triglycerides, aspartate transaminase, alanine transaminase, triglycerides, gamma-glutamyl transpeptidase, and lactate dehydrogenase). We utilized the most current laboratory data within a three-month interval preceding or after the CT scan.

### 2.2. CT Protocol and Image Analysis

**Figure 1:**
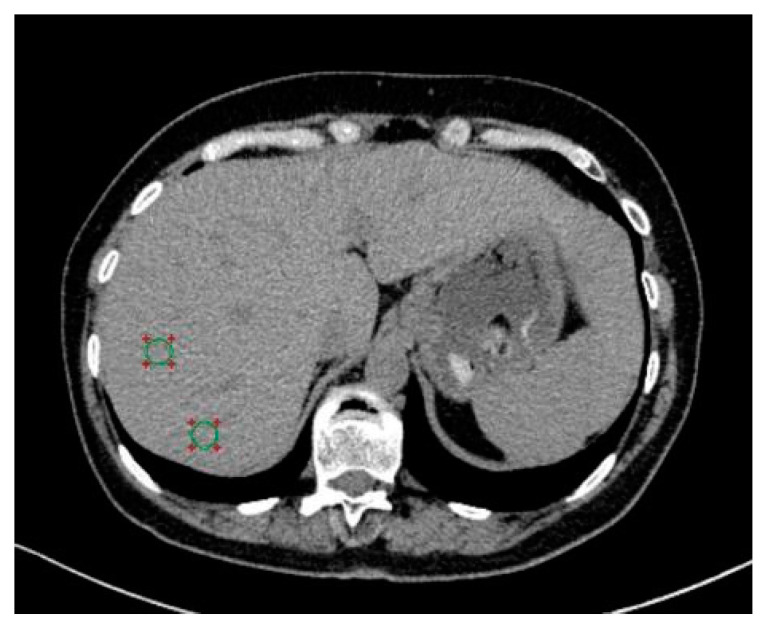
Showing a transverse non-enhanced CT scan with an example of where an ROI should be placed to evaluate attenuation. Two ROIs are positioned in the liver segments VII and VIII, represented by the two green circles.

An unenhanced abdominal CT examination was performed on a 64-slice MDCT (Somatom Definition, Siemens Medical Solutions) at 120 kVp during a single breath hold. An automated modulated tube current with a maximum setting of 200 mAs and a detector configuration of 241.2 mm was used. All unenhanced images were reconstructed at contiguous 3-mm intervals. The diagnostic criteria for hepatic steatosis on unenhanced CT were hypoattenuation of liver parenchyma with an absolute density of less than 48 HU, which was proposed in the previous literature [22,23,24]. By averaging the attenuation values (HU) for eight 1.5 cm^2^ circular ROIs positioned in separate axial slices in hepatic segments V, VI, VII, and VIII in accordance with the Couinaud technique, hepatic attenuation was calculated (Figure 1). Care was taken to examine representative regions of liver parenchyma while avoiding focal areas of fatty liver, fatty sparing, or any visible arteries. A radiologist blinded to the laboratory and clinical data analyzed the images.

### 2.3. Abdominal Adipose Tissue Measurement

On the abdominal CT scans, the depth of subcutaneous fat and the WC were determined. WC was measured at the level of the umbilicus. Three locations were used to measure the depth of subcutaneous fat: infraumbilical at the level of the iliac crest, right, and left midclavicular lines at the level of the umbilicus.

### 2.4. Statistical Analysis

Depending on their distribution, parameters are presented as the means and standard deviations, medians, and interquartile ranges, or counts and proportions. Continuous variables were compared using unpaired t-tests and Mann–Whitney U-tests. The χ^2^ test was used to compare categorical variables. To evaluate the correlation between abdominal adipose tissue measurements and liver attenuation values, Pearson and Spearman correlation coefficients were applied. Standard recognized criteria of none (0.0–0.1), weak (0.1–0.3), moderate (0.3–0.5), and high (0.5–1.0) correlation strengths were utilized [25]. The receiver operating characteristic (ROC) curve analysis was used to determine the cut-off values for all diagnostic tools, instruments, and laboratory findings to evaluate their predictive potential. Based on the Youden index (Youden Index = Sensitivity + Specificity 1, ranging from 0 to 1), the diagnostic tools cut-off values were determined using the ROC curve analysis. An evaluation for systematic bias was performed using the Bland and Altman method. For the statistical tests, a 5% (*p* < 0.05) level of significance was applied. Statistical analysis was performed using SPSS for Mac OS version 28.0.1.0 (SPSS, Inc., Chicago, IL, USA).

## 3. Results

Of the 119 individuals included, 66 (55%) were male. The average age was 54.54 +/− 12.90 years (range 20–85). Hepatic steatosis was present in 76 patients (63%; liver HU: 35 +/− 9.2).

**Figure 2:**
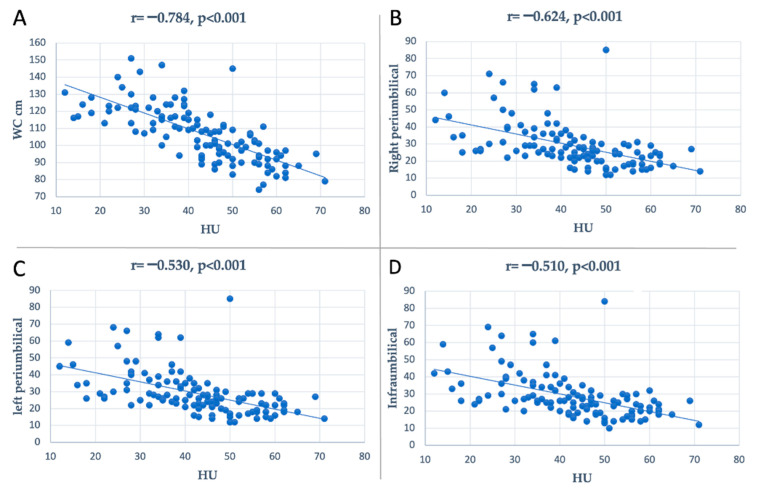
Plot representation of the dispersion of data of the correlation between abdominal anthropometric measurements and HU density; (**A**): correlation between WC and HU density; (**B**): correlation between right periumbilical subcutaneous fat thicknesses and HU density; (**C**): correlation between left periumbilical subcutaneous fat thicknesses and HU density; (**D**): correlation between infraumbilical subcutaneous fat thicknesses and HU density; WC, waist circumference.

Comparing patients with or without NAFLD, anthropometric measurements of obesity, including WC, infraumbilical, right paraumbilical, and left paraumbilical subcutaneous fat thicknesses, were significantly higher in individuals with steatosis (*p* < 0.0001), as shown in Table 1. Additionally, patients with NAFLD had significantly higher levels of triglycerides (*p* = 0.0005), GGT (*p* < 0.0001), and LDH (*p* = 0.001). The prevalence of type 2 diabetes was significantly higher in NAFLD patients (*p* = 0.0003). Among all patients, WC (r = −0.78, *p* < 0.0001), infraumbilical subcutaneous fat thicknesses (r = −0.51, *p* < 0.0001), right paraumbilical subcutaneous fat thicknesses (r = −0.62, *p* < 0.0001), and left paraumbilical subcutaneous fat thicknesses (r = −0.53, *p* < 0.0001) had a high inverse correlation with the liver attenuation values (Figure 2).

**Figure 3:**
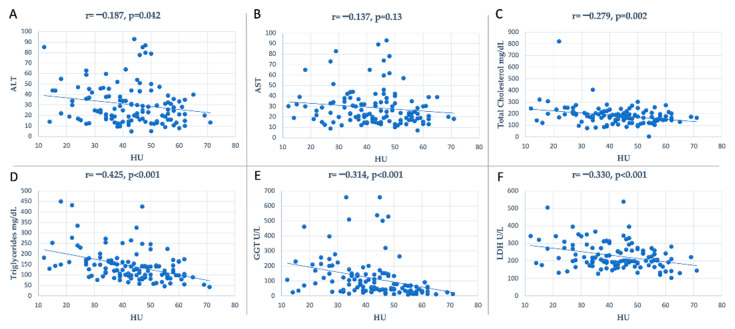
Plot representation of the dispersion of data of the correlation between laboratory parameters and HU density; (**A**): correlation between ALT and HU density; (**B**): correlation between AST and HU density; (**C**): correlation between total cholesterol and HU density; (**D**): correlation between triglycerides and HU density; (**E**): correlation between GGT and HU density; (**F**): correlation between LDH and HU density; ALT, alanine transaminase; AST, aspartate transaminase; GGT, gamma-glutamyl transpeptidase; LDH, lactate dehydrogenase.

Furthermore, the levels of triglycerides (r = −0.42, *p* < 0.001), gamma-glutamyl transpeptidase (r = −0.31, *p* < 0.001), lactate dehydrogenase (r = −0.33, *p* < 0.001) had a moderate inverse correlation with the liver attenuation values. The levels of total cholesterol (r = −0.27, *p* = 0.002) and alanine transaminase (r = −0.18, *p* = 0.04) demonstrated a weak inverse correlation with the liver attenuation values, as we see in Figure 3. Of note, WC was moderately correlated with the levels of triglycerides (r = 0.33, *p* = 0.0002) and total cholesterol (r = 0.23, *p* = 0.009).

**Figure 4:**
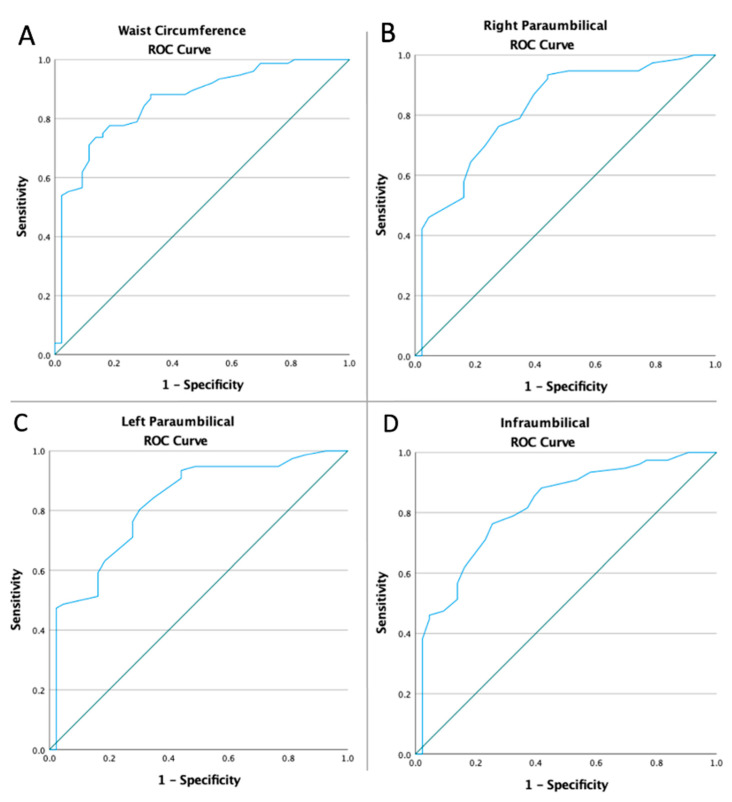
ROC Curve analyzed the NAFLD risk in terms of the abdominal anthropometric measurements (**A**): waist circumference (AUC: 0.859, *p* < 0.0001), (**B**): right paraumbilical (AUC: 0.819, *p* < 0.0001), (**C**): left paraumbilical (AUC: 0.821, *p* < 0.0001), and (**D**): infraumbilical (AUC: 0.816, *p* < 0.0001).

**Figure 5:**
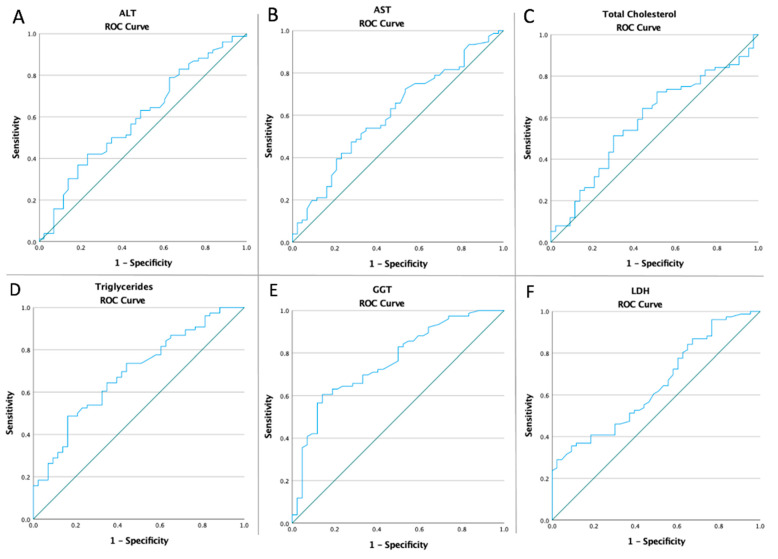
ROC Curve analyzed the NAFLD risk in terms of the laboratory parameters (**A**): ALT (AUC: 0.596, *p* = 0.083), (**B**): AST (AUC: 0.607, *p* = 0.054), (**C**): total cholesterol (AUC: 0.584, *p* = 0.127), (**D**): triglycerides (AUC: 0.690, *p* = 0.001), (**E**): GGT (AUC: 0.759, *p* < 0.0001), and (**F**): LDH (AUC: 0.646, *p* = 0.008); ALT, alanine transaminase; AST, aspartate transaminase; GGT, gamma-glutamyl transpeptidase; LDH, lactate dehydrogenase.

Receiver operating characteristic curves of all abdominal anthropometric measurements and laboratory parameters were computed in order to assess if the baseline values of all diagnostic tools and laboratory findings were predictive of the risk of NAFLD (Figure 4 and Figure 5). Table 2 displays the optimal cut-off value calculated using Youden’s index, the areas under the curve (AUC), sensitivity, and specificity of all diagnostic tools and laboratory findings analyzed.

To identify the predictive factors and diagnostic tools in terms of NAFLD risk, we performed multivariate analyses. As we see in Table 3, the presence of T2D (OR: 2.40, *p* = 0.04), WC (OR: 11.45, *p* < 0.001), right paraumbilical (OR: 10.09, *p* < 0.001), left paraumbilical (OR: 2.81, *p* = 0.01), and infraumbilical (OR: 3.06, *p* = 0.007) were strongly independent predictors of NAFLD risk. Moreover, regarding the laboratory parameters, only GGT (OR: 2.84, *p* = 0.009) is a predictor of NAFLD risk. The rest of the analyzed variables are presented in Table 3.

**Figure 6:**
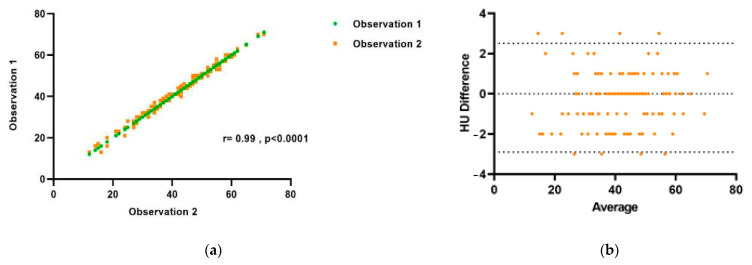
Results of Intra-observer reproducibility testing for liver HU measurement, shown using Pearson correlation coefficient (**a**) and Bland–Altman analysis (**b**).

The correlation between intra-reader variability and liver attenuation measurements was found to be excellent. The 95% confidence interval for intra-reader measures of liver HU density ranged from −2.89 to 2.51, and the mean difference was 0.2 (Figure 6).

## 4. Discussion

We carried out this study to better understand the relationship between WC, subcutaneous fat depth, and the diagnosis of non-alcoholic liver disease. As expected, NAFLD was strongly correlated with WC and subcutaneous fat depth measurements, which is in line with previous research findings [26,27,28,29,30], suggesting that as liver attenuation values decrease, anthropometric values assessing obesity increase. In addition, we discovered a correlation between triglyceride and total cholesterol levels and liver attenuation values, indicating that the higher the lipid levels, the more intense the CT-determined steatosis.

Past attempts at NAFLD evaluation were limited by the inclusion of non-specific markers as surrogates for steatosis, such as high ALT levels [31]. On the other hand, NAFLD cohorts based on liver biopsy are generally smaller in size and do not represent an asymptomatic population [20,32,33]. Apart from the associated risks and invasiveness, sample error and cost would also limit a large cohort based on biopsy investigation. In addition to CT, ultrasonography and MR imaging can be used to identify hepatic steatosis non-invasively. Ultrasound assessment, in contrast, is operator-dependent [34]. A variety of improved MR methods that can precisely evaluate hepatic fat content have recently been developed [20,32,33,35]. However, large-scale implementation would be constrained by availability and cost. In the presence of hepatic iron, dual-energy CT may also be helpful for quantifying hepatic fat [35,36]. The efficacy of measuring liver attenuation on unenhanced CT for detecting hepatic steatosis was recognized very early in CT’s clinical application [37]. The quantification of hepatic steatosis is essential because it provides information regarding the severity of the disease. The measurement of the liver parenchyma’s attenuation value, expressed in HU, serves as the foundation for the CT assessment of hepatic steatosis. It has been established that the degree of attenuation values’ reduction is related to the severity of hepatic steatosis [38]. The attenuation value of liver parenchyma decreases because fat has an attenuation value that is generally about −100 HU, much lower in comparison to soft tissue, which is generally about 30–40 HU. It is well known that the degree of hepatic steatosis detected on histopathologic examination presents a substantial association with the estimated absolute attenuation value of liver parenchyma on an unenhanced CT scan. Unenhanced CT scans are preferable for assessing hepatic steatosis because scan delays, altered organ perfusion, contrast type, contrast dose, and different injection protocols can affect the liver parenchyma’s attenuation value after contrast injection, despite several studies suggesting that contrast-enhanced CT images could also be used for the assessment of hepatic steatosis while offering relatively similar diagnostic accuracy to unenhanced CT scans [15,38,39]. Many studies have now been conducted to investigate the utility of CT in the context of NAFLD. The spleen has typically been employed as an internal reference standard in examinations, with the liver’s attenuation value being subtracted from it or the result being reported as a liver/spleen attenuation ratio. However, this method may be limited by the presence of occasional aberrant splenic attenuation. There is data that suggests liver attenuation alone on unenhanced CT, without splenic comparison, is a reliable indicator of the amount of hepatic fat [40,41]. Although there are drawbacks, such as radiation exposure, CT has faster data collection and is less expensive than MR. In a recent study, liver-fat measurements using magnetic resonance spectroscopy (MRS) and non-contrast-enhanced CT imaging showed a good correlation, especially in individuals with greater fat content [42]. Unenhanced CT only offers a 50% sensitivity and a 77% specificity for the detection of hepatic steatosis (≥5% steatosis at histopathology) [23]. Higher grades of steatosis (≥30% steatosis at histopathology) resulted in enhanced sensitivity and specificity (73% and 91%, respectively) [23].

Prior to the age of 50, the frequency of NAFLD rises, especially in males [43,44]. Men have a larger NAFLD risk than women in populations over 50, although postmenopausal women have the same risk as males their age [43,44]. Beyond the age of 50 for males and 70 for women, the prevalence of NAFLD declines [44]. The majority of NAFLD patients are asymptomatic, and frequently, when patients are assessed for another underlying condition, it is noticed incidentally that they have hepatomegaly or altered biochemical liver function tests [10,45]. Some patients have vague symptoms such as exhaustion, discomfort in the abdomen, or pain in the right upper quadrant. The majority of patients have a mild transaminase increase, and the typical ALT/AST ratio is less than one [46]. AST, ALT, and GGT are released into the blood by damaged liver cells, increasing plasma levels and indicating liver injury. These enzymes are sensitive markers of liver injury caused by a variety of diseases, but they are not particular markers for hepatic fat accumulation [47]. Gamma-glutamyl transpeptidase and alkaline phosphatase often express fluctuating elevations [46]. Once hepatitis C and other chronic illnesses have been ruled out, NAFLD is most frequently responsible for persistently unexplained elevated ALT levels [48]. Up to 80% of people may have fasting hypertriglyceridemia, and up to 50% may develop diabetes or glucose intolerance [10]. There may be an increase in serum ferritin and transferrin saturation along with an increase in iron in the liver [10]. 10% to 25% of NAFLD patients have been shown to have antinuclear antibodies [10,49]. Lactate production is significantly increased in hepatic steatosis [5]. The activity of acetylated LDH-B is decreased, impairing hepatocytes’ capacity to dispose of lactate and resulting in lactate buildup [50]. Increased lactate not only worsens hepatic steatosis but also raises the acetylation of histone H3K9 by decreasing the activity of nuclear histone deacetylase (HDAC), increasing fatty acid uptake, and the expression of genes involved in lipogenesis [5,50]. One constant feature of NAFLD is dyslipidemia. The liver produces and clears all forms of lipoprotein particles, making it essential for lipoprotein metabolism. NAFLD metabolic dysfunction is intimately connected to changes in lipoprotein metabolism and structure [51]. Generally, hepatocytes have a total lipid content of 4–7%, with TG making up around half of it [52]. An essential factor in the onset of NAFLD is the buildup of TG in liver cells. The amount of fat mass and stored fat grows with overeating, especially the VAT that provides fatty acids to the liver. If an excess of fatty acids is present, it can aggravate steatosis, produce reactive oxygen species, affect lipid metabolism, and cause injury to the liver [53,54,55]. Moreover, abnormal adipocytokines in the accumulating VAT enhance hepatic steatosis and the development of proinflammatory macrophages, all of which are linked to the emergence of NASH [53,56]. Subcutaneous adipose tissue (SAT) also plays a role in the development of NAFLD. Liver fat content and the number of macrophages in the SAT are correlated. The quantity of liver fat and the histological characteristics of NAFLD are both correlated with the expression of gene products that modulate inflammation in the SAT. Gene expression patterns in the VAT and SAT show that both tissues support the pathological development of NAFLD by employing similar pathways [56,57]. The adipose tissue in the lower body may serve as a storage site for fat, preventing the deposition of ectopic fat in the muscles and liver. This is because it has a higher lipoprotein lipase activity and a slower rate of fatty acid turnover compared to the adipose tissue in the abdominal region [58]. As a result, monitoring abdominal fat accumulation is crucial for NAFLD screening and prevention. In clinical practice, measuring waist circumference is a generally accepted and inexpensive procedure that provides a reliable indicator of the amount of visceral fat present in the body [59]. According to Jia et al., WC had the highest accuracy in predicting visceral obesity when compared to the waist-to-hip ratio (WHR) and body mass index (BMI) [60]. For assessing abdominal fat, CT has been recognized as an accurate and reliable method [61]. Even though many variables in this study had a correlation with NAFLD and the accuracy of predicting NAFLD was excellent, the abdominal anthropometric measurement parameters performed the best.

The diagnosis of NAFLD is clinically significant due to the substantial correlation between NAFLD and the metabolic syndrome, diabetes, and cardiovascular diseases as well as increasing data that suggests that the development of NASH may not be as uncommon an occurrence as previously thought [11]. The American Diabetes Association recommends regular ultrasonography screening for liver fibrosis and non-alcoholic steatohepatitis in individuals with type 2 diabetes and a fatty liver [62]. Similarly, the European Associations for the Study of Liver, Diabetes and Obesity recommend screening patients at high risk for NAFLD by liver enzyme assessment and/or abdominal ultrasonography [63]. Furthermore, given the high incidence of obesity and the metabolic syndrome, abdominal imaging cannot be used to screen all individuals at risk for NAFLD because this would likely overload imaging services [64]. As a result, a simplified approach for screening individuals with a high NAFLD risk is needed.

A range of pharmacological therapies, including antidiabetic, anti-obesity, antioxidants, and cytoprotective agents, have been applied clinically in the therapy of NAFLD [65]. There are several potential pharmacological targets and growing therapeutics with selective mechanisms available at the moment: fibrosis-targeted therapies, oxidative stress-targeted therapies, apoptosis-targeted therapies, inflammation-targeted therapies, and metabolic-targeted therapies [65].

Our research has several limitations. NAFLD was diagnosed in our study using CT, which is insensitive to mild steatosis [24]. As a result, the outcomes of mild NAFLD patients were misclassified. Furthermore, when fibrosis progresses, steatosis decreases, leading to misdiagnosis. Due to a lack of histologic data, we were unable to assess non-alcoholic steatohepatitis. Future research using MRI methods for quantifying hepatic steatosis may therefore assist in clearing this aspect and supporting our findings.

## 5. Conclusions

Health services are facing a serious challenge from the global increase in obesity. NAFLD has become the most prevalent liver condition, and its incidence is closely correlated with obesity levels. To develop preventive and treatment strategies, a thorough knowledge of the processes underlying disease development is urgently needed.

WC, the depth of subcutaneous fat, triglycerides, gamma-glutamyl transpeptidase, lactate dehydrogenase, and type 2 diabetes were significantly associated with the occurrence of NAFLD as determined by non-contrast-enhanced CT. A significant inverse correlation was identified between abdominal adipose tissue measurements and the liver attenuation values. In addition, our data indicate that triglycerides and total cholesterol are also inversely correlated with the liver attenuation values. Clinical and laboratory profile abnormalities that indicate metabolic syndrome may be associated with the presence of moderate or severe hepatic steatosis detected by non-enhanced CT criteria.

## Figures and Tables

**Table 1:** Comparison between individuals with and without liver steatosis.

	No Hepatic Steatosis	Hepatic Steatosis	*p*-Value
N	43	76	
Age	53.67 ± 14.58	58.16 ± 11.64	0.068
Gender			0.2
Female	22 (51.16%)	31 (40.79%)	
Male	21 (48.84%)	45 (59.21%)	
Abdominal anthropometric measurements			
WC (cm)	95.09 ± 12.17	114 ± 14.25	**<0.0001**
Suprapubically	20 [16–25]	20 [16–25]	**<0.0001**
Right paraumbilical	20 [16–25]	29 [25–37]	**<0.0001**
Left paraumbilical	19 [17–26]	29 [25–37]	**<0.0001**
Laboratory parameters			
Total cholesterol (mg/dL)	165 [130–196.1]	183.6 [145.5–218.3]	0.1275
TG(mg/dL)	101.1 [84–141.9]	142.8 [99.50–180.1]	**0.0005**
AST (U/I)	20.20 [16–30.1]	26 [18.25–37.65]	0.053
ALT (U/I)	21.60 [16.93–43.93]	27.65 [13.40–33.40]	0.08
GGT (U/l)	40 [19–69.25]	108 [43–186.8]	**<0.0001**
LDH (U/l)	199 [163–241]	218 [189–300]	**0.001**
Co-morbidities			
T2D	6 (14.29%)	36 (85.71%)	**0.0003**
Hypertension	24 (35.83%)	43 (64.18%)	0.9

For continuous variables, the results are expressed as mean (SD) or median [interquartile range], and n (%) for categorical variables. N, number of individuals; WC, waist circumference; TG: triglycerides; ALT, alanine transaminase; AST, aspartate transaminase; GGT, gamma-glutamyl transpeptidase; LDH, lactate dehydrogenase; T2D, type 2 diabetes. Bold indicates statistical significance.

**Table 2:** ROC curves, ideal cut-off value, AUC, and prediction accuracy of abdominal anthropometric measurements and laboratory parameters and risk of NAFLD.

Variables	Cut-Off	AUC	Std. Error	95% CI	Sensitivity	Specificity	*p*-Value
**Abdominal anthropometric measurements**							
**WC**	101.5	0.859	0.035	0.790–0.929	77.6%	76.7%	**<0.0001**
**Right Paraumbilical**	22.5	0.819	0.041	0.740–0.899	86.8%	60.5%	**<0.0001**
**Left Paraumbilical**	23.5	0.821	0.040	0.742–0.900	80.3%	69.8%	**<0.0001**
**Infraumbilical**	24.5	0.816	0.041	0.736–0.895	76.3%	74.4%	**<0.0001**
**Laboratory parameters**							
**ALT**	-	0.596	0.054	0.489–0.702	-	-	0.083
**AST**	-	0.607	0.054	0.502–0.712	-	-	0.054
**Total cholesterol**	-	0.584	0.054	0.478–0.691	-	-	0.127
**Triglycerides**	124.44	0.690	0.049	0.593–0.787	60.5%	67.4%	**0.001**
**GGT**	82.0	0.759	0.046	0.669–0.849	60.5%	85.7%	**<0.0001**
**LDH**	245.5	0.646	0.051	0.546–0.746	40.8%	81.4%	**0.008**

WC, waist circumference; ALT, alanine transaminase; AST, aspartate transaminase; GGT, gamma-glutamyl transpeptidase; LDH, lactate dehydrogenase. Bold indicates statistical significance.

**Table 3:** Multivariate analyses of the age, risk factors, abdominal anthropometric measurements, laboratory parameters, and the NAFLD risk.

NAFLD			
	OR	95% CI	*p*-Value
**AGE**	1.02	0.99–1.05	0.07
**T2D**	2.40	1.03–5.56	**0.04**
**ABDOMINAL ANTHROPOMETRIC MEASUREMENTS**			
**WC**	11.45	4.70–27.88	**<0.001**
**RIGHT PARAUMBILICAL**	10.09	4.09–24.91	**<0.001**
**LEFT PARAUMBILICAL**	2.81	1.21–6.51	**0.01**
**INFRAUMBILICAL**	3.06	1.35–6.95	**0.007**
**LABORATORY PARAMETERS**			
**TRIGLYCERIDES**	1.21	0.57–2.56	0.37
**GGT**	2.84	1.30–6.23	**0.009**
**LDH**	2.03	0.87–4.74	0.09

WC, waist circumference; GGT, gamma-glutamyl transpeptidase; LDH, lactate dehydrogenase; T2D, type 2 diabetes. (Bold indicates statistical significance).

## Data Availability

Not applicable.

## References

[1] Angulo P. (2002). Nonalcoholic Fatty Liver Disease. N. E. J. Med..

[2] Younossi Z.M., Koenig A.B., Abdelatif D., Fazel Y., Henry L., Wymer M. (2016). Global epidemiology of nonalcoholic fatty liver disease-Meta-analytic assessment of prevalence, incidence, and outcomes. Hepatology.

[3] Estes C., Razavi H., Loomba R., Younossi Z., Sanyal A.J. (2018). Modeling the epidemic of nonalcoholic fatty liver disease demonstrates an exponential increase in burden of disease. Hepatology.

[4] Younossi Z.M., Anstee Q.M., Marietti M., Hardy T., Henry L., Eslam M., George J., Bugianesi E. (2017). Global burden of NAFLD and NASH: Trends, predictions, risk factors and prevention. Nat. Rev. Gastroenterol. Hepatol..

[5] Lu Q., Tian X., Wu H., Huang J., Li M., Mei Z., Zhou L., Xie H., Zheng S. (2021). Metabolic Changes of Hepatocytes in NAFLD. Front. Physiol..

[6] Stepanova M., Rafiq N., Makhlouf H., Agrawal R., Kaur I., Younoszai Z., McCullough A., Goodman Z., Younossi Z.M. (2013). Predictors of All-Cause Mortality and Liver-Related Mortality in Patients with Non-Alcoholic Fatty Liver Disease (NAFLD). Dig. Dis. Sci..

[7] Benedict M., Zhang X. (2017). Non-alcoholic fatty liver disease: An expanded review. World J. Hepatol..

[8] Nassir F., Rector R.S., Hammoud G.M., Ibdah J.A. (2015). Pathogenesis and Prevention of Hepatic Steatosis. Gastroenterol. Hepatol..

[9] Sheka A.C., Adeyi O., Thompson J., Hameed B., Crawford P.A., Ikramuddin S. (2020). Nonalcoholic Steatohepatitis. JAMA.

[10] Mirza M.S. (2011). Obesity, Visceral Fat, and NAFLD: Querying the Role of Adipokines in the Progression of Nonalcoholic Fatty Liver Disease. ISRN Gastroenterol..

[11] Lindenmeyer C.C., McCullough A.J. (2017). The Natural History of Nonalcoholic Fatty Liver Disease—An Evolving View. Clin. Liver Dis..

[12] Portillo-Sanchez P., Bril F., Maximos M., Lomonaco R., Biernacki D., Orsak B., Subbarayan S., Webb A., Hecht J., Cusi K. (2015). High Prevalence of Nonalcoholic Fatty Liver Disease in Patients With Type 2 Diabetes Mellitus and Normal Plasma Aminotransferase Levels. J. Clin. Endocrinol. Metab..

[13] Adams L.A., Lymp J.F., Sauver J.S., Sanderson S.O., Lindor K.D., Feldstein A., Angulo P. (2005). The Natural History of Nonalcoholic Fatty Liver Disease: A Population-Based Cohort Study. Gastroenterology.

[14] Chalasani N., Younossi Z., LaVine J.E., Charlton M., Cusi K., Rinella M., Harrison S.A., Brunt E.M., Sanyal A.J. (2018). The diagnosis and management of nonalcoholic fatty liver disease: Practice guidance from the American Association for the Study of Liver Diseases. Hepatology.

[15] Lee D.H. (2017). Imaging evaluation of non-alcoholic fatty liver disease: Focused on quantification. Clin. Mol. Hepatol..

[16] Keihani S., Hosseinpanah F., Barzin M., Serahati S., Doustmohamadian S., Azizi F. (2014). Abdominal obesity phenotypes and risk of cardiovascular disease in a decade of follow-up: The Tehran Lipid and Glucose Study. Atherosclerosis.

[17] Byrne C.D., Targher G. (2015). NAFLD: A multisystem disease. J. Hepatol..

[18] Pop R.-M., Pop M., Dogaru G.C., Bacarea V. (2013). A Web-based Nutritional Assessment Tool. Stud. Informatics Control.

[19] Zhou J.-H., Cai J.-J., She Z.-G., Li H.-L. (2019). Noninvasive evaluation of nonalcoholic fatty liver disease: Current evidence and practice. World J. Gastroenterol..

[20] Castera L., Friedrich-Rust M., Loomba R. (2019). Noninvasive Assessment of Liver Disease in Patients with Nonalcoholic Fatty Liver Disease. Gastroenterology.

[21] Sheng G., Lu S., Xie Q., Peng N., Kuang M., Zou Y. (2021). The usefulness of obesity and lipid-related indices to predict the presence of Non-alcoholic fatty liver disease. Lipids Health Dis..

[22] Jawahar A., Gonzalez B., Balasubramanian N., Adams W., Goldberg A. (2017). Comparison of correlations between lipid profile and different computed tomography fatty liver criteria in the setting of incidentally noted fatty liver on computed tomography examinations. Eur. J. Gastroenterol. Hepatol..

[23] Lee S.S., Park S.H., Kim H.J., Kim S.Y., Kim M.-Y., Kim D.Y., Suh D.J., Kim K.M., Bae M.H., Lee J.Y. (2010). Non-invasive assessment of hepatic steatosis: Prospective comparison of the accuracy of imaging examinations. J. Hepatol..

[24] Zeb I., Li D., Nasir K., Katz R., Larijani V.N., Budoff M.J. (2012). Computed Tomography Scans in the Evaluation of Fatty Liver Disease in a Population Based Study. Acad. Radiol..

[25] Cohen J. (1988). Statistical Power Analysis for the Behavioral Sciences.

[26] Wells M.M., Li Z., Addeman B., McKenzie C.A., Mujoomdar A., Beaton M., Bird J. (2016). Computed Tomography Measurement of Hepatic Steatosis: Prevalence of Hepatic Steatosis in a Canadian Population. Can. J. Gastroenterol. Hepatol..

[27] (2021). Association of abdominal subcutaneous fat thickness with hepatic steatosis, liver enzymes, and serum lipids in obese children. Arch. Argent. de Pediatr..

[28] Reis S.S., Callejas G.H., Marques R.A., Gestic M.A., Utrini M.P., Chaim F.D.M., Ramos A.C., Chaim E.A., Cazzo E. (2021). Correlation Between Anthropometric Measurements and Non-alcoholic Fatty Liver Disease in Individuals With Obesity Undergoing Bariatric Surgery: Cross-Sectional Study. Obes. Surg..

[29] Baek J., Jung S.J., Shim J.-S., Jeon Y.W., Seo E., Kim H.C. (2020). Comparison of Computed Tomography-based Abdominal Adiposity Indexes as Predictors of Non-alcoholic Fatty Liver Disease Among Middle-aged Korean Men and Women. J. Prev. Med. Public Health.

[30] Zhou K., Dodge J.L., Yuan L., Terrault N.A. (2021). Metabolic Risk Profiles for Hepatic Steatosis Differ by Race/Ethnicity: An Elastography-Based Study of US Adults. Dig. Dis. Sci..

[31] Dunn W., Xu R., Wingard D.L., Rogers C., Angulo P., Younossi Z.M., Schwimmer J.B. (2008). Suspected Nonalcoholic Fatty Liver Disease and Mortality Risk in a Population-Based Cohort Study. Am. J. Gastroenterol..

[32] Virarkar M., Szklaruk J., Jensen C.T., Taggart M.W., Bhosale P. (2021). What’s New in Hepatic Steatosis. Semin. Ultrasound CT MRI.

[33] Trujillo M.J., Chen J., Rubin J.M., Gao J. (2021). Non-invasive imaging biomarkers to assess nonalcoholic fatty liver disease: A review. Clin. Imaging.

[34] Strauss S., Gavish E., Gottlieb P., Katsnelson L. (2007). Interobserver and Intraobserver Variability in the Sonographic Assessment of Fatty Liver. Am. J. Roentgenol..

[35] Lee S.S. (2014). Radiologic evaluation of nonalcoholic fatty liver disease. World J. Gastroenterol..

[36] Fischer M.A., Gnannt R., Raptis D., Reiner C.S., Clavien P.-A., Schmidt B., Leschka S., Alkadhi H., Goetti R. (2011). Quantification of Liver Fat in the Presence of Iron and Iodine. Investig. Radiol..

[37] Bydder G.M., Kreel L., Chapman R.W., Harry D., Sherlock S., Bassan L. (1980). Accuracy of computed tomography in diagnosis of fatty liver. BMJ.

[38] Idilman I.S., Ozdeniz I., Karcaaltincaba M. (2016). Hepatic Steatosis: Etiology, Patterns, and Quantification. Semin. Ultrasound, CT MRI.

[39] Chung J., Park H.-S., Kim Y.-J., Yu M.-H., Park S., Jung S.-I. (2021). Association of Hepatic Steatosis Index with Nonalcoholic Fatty Liver Disease Diagnosed by Non-Enhanced CT in a Screening Population. Diagnostics.

[40] Kodama Y., Ng C.S., Wu T.T., Ayers G.D., Curley S.A., Abdalla E.K., Vauthey J.N., Charnsangavej C. (2007). Comparison of CT Methods for Determining the Fat Content of the Liver. Am. J. Roentgenol..

[41] Pickhardt P.J., Park S.H., Hahn L., Lee S.-G., Bae K.T., Yu E.S. (2011). Specificity of unenhanced CT for non-invasive diagnosis of hepatic steatosis: Implications for the investigation of the natural history of incidental steatosis. Eur. Radiol..

[42] Kramer H., Pickhardt P.J., Kliewer M.A., Hernando D., Chen G.-H., Zagzebski J.A., Reeder S.B. (2017). Accuracy of Liver Fat Quantification With Advanced CT, MRI, and Ultrasound Techniques: Prospective Comparison With MR Spectroscopy. Am. J. Roentgenol..

[43] Long M.T., Pedley A., Massaro J.M., Hoffmann U., Ma J., Loomba R., Chung R.T., Benjamin E. (2018). A simple clinical model predicts incident hepatic steatosis in a community-based cohort: The Framingham Heart Study. Liver Int..

[44] Bertolotti M., Lonardo A., Mussi C., Baldelli E., Pellegrini E., Ballestri S., Romagnoli D., Loria P. (2014). Nonalcoholic fatty liver disease and aging: Epidemiology to management. World J. Gastroenterol..

[45] Farrell G.C., Larter C.Z. (2006). Nonalcoholic fatty liver disease: From steatosis to cirrhosis. Hepatology.

[46] Mofrad P., Contos M.J., Haque M., Sargeant C., Fisher R.A., Luketic V.A., Sterling R.K., Shiffman M.L., Stravitz R.T., Sanyal A.J. (2003). Clinical and histologic spectrum of nonalcoholic fatty liver disease associated with normal ALT values. Hepatology.

[47] Yamakado M., Tanaka T., Nagao K., Imaizumi A., Komatsu M., Daimon T., Miyano H., Tani M., Toda A., Yamamoto H. (2017). Plasma amino acid profile associated with fatty liver disease and co-occurrence of metabolic risk factors. Sci. Rep..

[48] Sanyal A.J. (2002). AGA technical review on nonalcoholic fatty liver disease. Gastroenterology.

[49] Cotler S.J., Kanji K., Keshavarzian A., Jensen D.M., Jakate S. (2004). Prevalence and Significance of Autoantibodies in Patients With Non-Alcoholic Steatohepatitis. J. Clin. Gastroenterol..

[50] Wang T., Chen K., Yao W., Zheng R., He Q., Xia J., Li J., Shao Y., Zhang L., Huang L. (2020). Acetylation of lactate dehydrogenase B drives NAFLD progression by impairing lactate clearance. J. Hepatol..

[51] Deprince A., Haas J.T., Staels B. (2020). Dysregulated lipid metabolism links NAFLD to cardiovascular disease. Mol. Metab..

[52] Liu H., Li X., Han X., Zhang Y., Gu Y., Sun L., Han J., Tu Y., Bao Y., Bai W. (2022). Simple surrogate equations to predict controlled attenuation parameter values for screening non-alcoholic fatty liver disease in a Chinese population. Front. Med..

[53] Miyake T., Miyazaki M., Yoshida O., Kanzaki S., Nakaguchi H., Nakamura Y., Watanabe T., Yamamoto Y., Koizumi Y., Tokumoto Y. (2021). Relationship between body composition and the histology of non-alcoholic fatty liver disease: A cross-sectional study. BMC Gastroenterol..

[54] Rolo A.P., Teodoro J.S., Palmeira C.M. (2012). Role of oxidative stress in the pathogenesis of nonalcoholic steatohepatitis. Free. Radic. Biol. Med..

[55] Donnelly K.L., Smith C.I., Schwarzenberg S.J., Jessurun J., Boldt M.D., Parks E.J. (2005). Sources of fatty acids stored in liver and secreted via lipoproteins in patients with nonalcoholic fatty liver disease. J. Clin. Investig..

[56] Polyzos S.A., Kountouras J., Zavos C., Tsiaousi E. (2010). The role of adiponectin in the pathogenesis and treatment of non-alcoholic fatty liver disease. Diabetes Obes. Metab..

[57] Du Plessis J., Van Pelt J., Korf H., Mathieu C., Van Der Schueren B., Lannoo M., Oyen T., Topal B., Fetter G., Nayler S. (2015). Association of Adipose Tissue Inflammation With Histologic Severity of Nonalcoholic Fatty Liver Disease. Gastroenterology.

[58] Vega G.L., Adams-Huet B., Peshock R., Willett D., Shah B., Grundy S.M. (2006). Influence of Body Fat Content and Distribution on Variation in Metabolic Risk. J. Clin. Endocrinol. Metab..

[59] Ross R., Neeland I.J., Yamashita S., Shai I., Seidell J., Magni P., Santos R.D., Arsenault B., Cuevas A., Hu F.B. (2020). Waist circumference as a vital sign in clinical practice: A Consensus Statement from the IAS and ICCR Working Group on Visceral Obesity. Nat. Rev. Endocrinol..

[60] Jia W.-P., Lu J.-X., Xiang K.-S., Bao Y.-Q., Lu H.-J., Chen L. (2003). Prediction of abdominal visceral obesity from body mass index, waist circumference and waist-hip ratio in Chinese adults: Receiver operating characteristic curves analysis. Biomed. Environ. Sci..

[61] Rössner S., Bo W.J., Hiltbrandt E., Hinson W., Karstaedt N., Santago P., Sobol W.T., Crouse J.R. (1990). Adipose tissue determinations in cadavers--a com-parison between cross-sectional planimetry and computed tomography. Int. J. Obes..

[62] (2016). EASL–EASD–EASO Clinical Practice Guidelines for the management of non-alcoholic fatty liver disease. J. Hepatol..

[63] American Diabetes Association 4 (2018). Comprehensive Medical Evaluation and Assessment of Comorbidities: Standards of Medical Care in Diabetes—2019. Diabetes Care.

[64] Dowman J.K., Tomlinson J.W., Newsome P.N. (2010). Systematic review: The diagnosis and staging of non-alcoholic fatty liver disease and non-alcoholic steatohepatitis. Aliment. Pharmacol. Ther..

[65] Negi C.K., Babica P., Bajard L., Bienertova-Vasku J., Tarantino G. (2021). Insights into the molecular targets and emerging pharmacotherapeutic interventions for nonalcoholic fatty liver disease. Metabolism.

